# The Duties of a Sister-Tutor: The Strain of Constant Teaching

**Published:** 1919-07-19

**Authors:** 


*To the Editor of* THE HOSPITAL.

### The Duties of a Sister-Tutor: The Strain of Constant Teaching.

*To the Editor of* THE HOSPITAL.

SIR,—I feel it my duty to suggest that in arranging the duties of sister-tutors the matrons should consult with educational experts as to the number of hours any one woman can teach efficiently in a day, and also as to the amount of other work which should be expected of her. The appointment of sister-tutors is a comparatively new thing, and the work of such an official is considered by many to be absurdly easy and unexacting compared with that of a ward or other sister or assistant matron. As to preparation and revision of notes for lectures, reading to keep herself up to date, visits to the wards to keep in touch with the clinical work, these matters are simply not taken into consideration at all. I was a mistress in two well-known high schools before entering the nursing profession, and no mistress, then or now, would be asked to teach for more than fifteen hours a week with three, or, at most, four and a-half hours' supervision of pupils' preparations in addition, during which time many books could be corrected. There were no other duties at all, except, of course, the preparation of lessons, correcting of books, and keeping of registers. Saturday and Sunday were entirely free, and thirteen or fourteen weeks' or more holiday given each year—and school mistresses are not considered idle mortals. We often undertook voluntary duties, of course, in connection with the girls' recreations, but, then, we were not too tired to enjoy them ourselves.

During the war many senior sisters in civil hospitals have undertaken work that no one woman ought to attempt in normal times, in order that, as far as in us lay, the nurses of the future should not suffer, and yet our gallant men be properly nursed; but are we always to be bled like this? It looks rather like it to judge from the statement of duties expected in a new appointment. Senior sisters are not, as a rule, the grumblers of the profession. They are too much occupied with the affairs of others to think very much about their own grievances, and will go on as long as brain and nerves will respond to the calls made upon them. This is all very well, or, it is not, in purely administrative posts, but all experienced head mistresses know that in the hands of an overworked mistress teaching soon deteriorates into irritable cramming, hateful to the pupils, heartbreaking to the teacher. The preliminary school is not a farm of fresh, if not brilliant, children, accustomed to mental application and of about the same age and capacity. It is a medley of young women drawn from various ranks of society, their ages varying perhaps fifteen years and educational attainments beginning with the leaving examination of an elementary school and ending with a University graduate's. The task of keeping the class together seems sometimes well-nigh impossible, and can only be done by dint of much individual coaching. The constantly recurring examinations, the difficulty of obtaining books of reference, catering and housekeeping anxieties, the never-ending stream of fresh pupils, and, above all, the high standard of life and conduct to be upheld to them and strained after by them, become crushing in time to the most buoyant and enthusiastic spirit. I feel sure, however, that if the difficulties are once understood that they will be considered and provided for, so that the working years of the more highly educated nurses need not be ruthlessly shortened by overstrain.

I am, Sir, yours faithfully,

July 13, 1919. A SISTER-TUTOR.

